# MicroRNA-23b: Roles, functions and mechanisms in tumor

**DOI:** 10.1016/j.gendis.2025.101853

**Published:** 2025-09-18

**Authors:** Xinyu Cai, Xueer Zheng, Zhenru Wang, Jiahua Mao, Minhe Shen, Shanming Ruan

**Affiliations:** The First Affiliated Hospital of Zhejiang Chinese Medical University (Zhejiang Provincial Hospital of Chinese Medicine), Hangzhou, Zhejiang 310006, China

**Keywords:** Gene expression regulation, MicroRNA, miR-23b, Role, Tumor

## Abstract

MicroRNAs are a class of non-coding short-stranded RNAs with important biological roles as post-transcriptional regulators of gene expression, involved in a variety of biological processes, such as cell growth, apoptosis, and angiogenesis. miR-23b, a well-studied miRNA, is aberrantly expressed in a variety of cancers. In this paper, we used the literature review method to sort out the biological roles and regulation of miR-23b in different types of cancers, including digestive system cancers, reproductive system cancers, head and neck cancers, genitourinary system cancers, and lung cancers. The data show that miR-23b can target both oncogenes and tumor suppressors, and its expression regulation in different types of cancers shows heterogeneity, which mainly acts through affecting signaling pathways, such as Wnt/β-catenin for tumorigenesis, and apoptotic proteins, such as BCL2 or oncogenes. The expression of miR-23b is closely related to the overall survival rate, disease-free survival rate, and prognosis in many cancers. Meanwhile, various *in vivo* and *ex vivo* studies have shown that miR-23b is a potential therapeutic target for cancer. It is important to understand the relationship between miR-23b and cancer development. miR-23b has the potential to serve as a clinically relevant molecular biomarker for cancer prognosis/diagnosis as well as a potential therapeutic target.

## Introduction

MicroRNAs (miRNAs), a class of endogenous non-coding RNAs and ∼19–25 nucleotides in length, are widely conserved in eukaryotes and primarily regulate gene expression post-transcriptionally.^1^^,^^2^ The latest database updates show that over 30,000 mature miRNAs have been identified in humans, predominantly generated through Dicer enzyme-mediated cleavage of ∼70-nucleotide hairpin-structured pre-miRNAs.^3^ These molecules are essential for cell proliferation, differentiation, and metabolism,^4^ with dysregulated expression linked to major human diseases, such as cancer, neurological disorders, and cardiovascular diseases.^5^, ^6^, ^7^

Since Calin’s team first reported miR-15a/16 cluster alterations in leukemia in 2002,^8^ cancer miRNA research has grown exponentially. Their aberrant expression in tumors has spurred their use as diagnostic/prognostic biomarkers and potential therapeutic targets. miRNAs influence key oncogenic processes, including proliferation, apoptosis, invasion, metastasis, and angiogenesis, with functions often bidirectional. For example, miR-17-92 cluster drove lung cancer and breast cancer progression,^9^ while miR-155 overexpression promoted malignancy across tumor types. Conversely, miR-34a and miR-126 suppressed tumorigenesis by inhibiting oncogenes.^10^ The 2024 Nobel Prize in Physiology or Medicine, awarded for miRNA discovery and post-transcriptional regulation, underscored this field’s research prominence and scientific significance.

Mature miRNAs regulate gene expression by binding to the 3′ UTR of target mRNAs via base pairing (RNAi).^4^ RNAi and delivery vectors are widely used in cancer therapy to silence oncogenes. RNAi (including miRNA-mediated pathways) modulates gene expression for disease treatment.^11^ Small interfering RNA (siRNA), a key RNAi mediator, efficiently silences genes, such as C-X-C motif chemokine receptor 4 (CXCR4) siRNA, via dextran-arginine nanoparticles inhibiting metastasis in preclinical models.^12^^,^^13^ Chemical modifications and advanced vectors enhance siRNA stability and uptake and reduce immunogenicity/off-target effects for effective cancer therapy. DNA/RNA delivery technologies (viral/non-viral vectors like lipid/polymer nanoparticles) show broad prospects,^14^, ^15^, ^16^ providing a basis for RNAi clinical application.

Among numerous miRNAs, miR-23b (NCBI; Table S1; Fig. S1) originates from human chromosome 9 (9q22.32) as part of the miR-23b/27b/24-1 cluster. Its hairpin pre-miR-23b yields functional mature strands miR-23b-3p and miR-23b-5p.^17^ Regulated by transcription factors like nuclear factor kappa B (NF-κB), p53, and c-Myc,^18^^,^^19^ miR-23b controls cell proliferation, migration, and adhesion. Dysregulation links to cancer, immune disorders, and thyroid dysfunction.^20^, ^21^, ^22^ In cancer, it inhibits gastrointestinal tumor invasion by targeting oncogenes (*e.g.*, NCAPG),^23^ yet promotes breast cancer via suppressing tumor suppressors.^24^ This functional duality makes it a key focus for tumor precision therapy.

Consequently, this work summarized and categorized the significance of miR-23b across various malignancies, aiming to elucidate its regulation patterns in distinct cancer types. Further investigation into the role of miR-23b in tumor suppression or promotion will establish a theoretical framework for cancer treatment.

## Analysis of research hotspots and trends of miR-23b

A systematic search of the Web of Science Core Collection (WoSCC) was conducted using the query “TS=(cancer OR tumor OR neoplasm OR carcinoma) AND TS=(mir-23b)”, covering the SCI-EXPANDED database from 1975 to present with English articles as the document type. A total of 622 papers were downloaded on November 23, 2024. After manual screening to remove redundant materials and exclusion of literature lacking author information or keyword fields, 281 works were retained for knowledge graph construction (Fig. S2). Data quality was enhanced via Co-Occurrence (COOC) software for cleansing and deduplication prior to graph building.

Professional bibliometric tools (CiteSpace 6.1.R3, VOSviewer 1.6.20, COOC14.9) were applied. Figure S3A depicts a keyword co-occurrence network: colored nodes denote clustering modules, line thickness indicates co-occurrence frequency, and node size reflects keyword prevalence.^25^ miR-23b research clustered into five cancer modules, with hotspots including miR-23b-3p, apoptosis, biomarkers, exosomes, prognosis, gene expression, and digestive/urological/gynecological cancers.

Leveraging CiteSpace for temporal analysis (due to VOSviewer limitations), the top 25 terms (Fig. S3B) showed “hepatocellular carcinoma” most frequent, followed by “clinicopathological feature” and “immune infiltration”. “Proliferation” had the longest top-25 tenure, while “renal cancer” emerged first. Current keywords like “colorectal cancer”, “extracellular vesicles”, and “immune infiltration” suggest ongoing focus on disease prognosis and specific cancer types.

## Role and function of miR-23b in tumors

**Figure 1:**
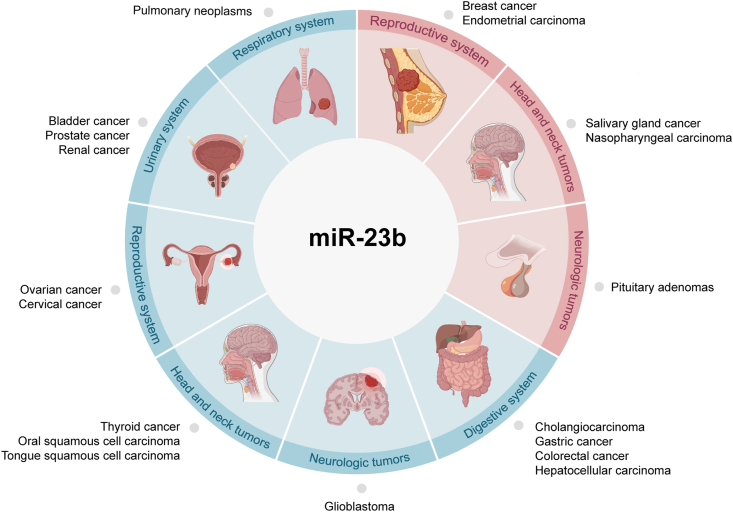
The role of miR-23b in tumors. miR-23b acts as a tumor suppressor in tissues and organs, such as the liver, stomach, colorectum, lung, and ovary (blue marker), and exerts pro-carcinogenic effects in breast, pituitary, and cervix (red marker). Created with BioRender.

Like other miRNAs, miR-23b is up- or down-regulated in normal tissue. It functions as a negative regulator of tumor suppressor proteins, which has an oncogenic function (onco-miR), as well as a tumor suppressor miRNA (ts-miR), which targets oncogenes to reduce the invasiveness of tumor cells. Below and in Figure 1, we provide an overview of current research on the dual function of miR-23b in cancer.

## Tumors of the digestive system

### Colorectal cancer

Colorectal cancer (CRC) is the second most prevalent cause of cancer-related deaths globally and the third most common type of cancer overall.^26^ It is seen as a global health issue, and novel therapeutic approaches are desperately needed to address it.

miR-23b expression is linked to poor prognostic features, such as low tumor differentiation, high TNM stage, and distant metastasis, and there is growing evidence that up-regulating it may prevent the development of CRC.^27^^,^^28^ According to an intriguing study conducted on migrating and non-migrating cell lines, miR-23b/27b/24 clusters were elevated in migrating cell lines. This suggests that miR-23b/27b/24 clusters may encourage cell migration by targeting forkhead box P2 (FOXP2).^29^ The role of miR-23b in ubiquitous CRC cells and individual miR-23b is unknown, even though miR-23b is probably inadequate in this study. The expression of miR-23b in CRC cells may be closely related to epigenetic regulation. Kou et al^30^ showed that increased miR-23b methylation of CRC cells directly suppressed its promoter transcription, reducing miR-23b synthesis. The promoter methylation status was inversely correlated with miR-23b expression, and demethylating agents restored it. Insufficient miR-23b expression allowed tumor cells to proliferate and migrate, which promoted CRC development. However, reducing promoter methylation increased miR-23b expression, which targeted related genes, including phosphodiesterase 7A (*PDE7A*) gene, to inhibit cell motility and invasion, and suppressed tumor growth.

According to Grisard et al,^31^ miR-23b interacted with BTB/POZ domain-containing protein 7 (BTBD7) to operate as an anti-metastatic molecule in CRC, with various functions depending on when the tumor metastasized. By targeting nuclear factor erythroid 2-like 3 (NFE2L3), structural maintenance of chromosomes 1 (SMC1A), KLF transcription factor 3 (KLF3), and other proteins, miR-23b-3p also contributed to the development of CRC by influencing the biological behaviors of CRC cells, including invasion, migration, and proliferation.^27^^,^^32^ Furthermore, Li et al^33^ employed informatics prediction, dual luciferase assays, and cell counting kit-8 (CCK8) for experimental validation, asserting that miR-23b-3p targeted the non-SMC condensin I complex subunit G (NCAPG) by modulating the phosphoinositide 3-kinase (PI3K)/protein kinase B (AKT) signaling pathway, thereby promoting CRC cell proliferation and inhibiting apoptosis.

### Gastric cancer

Gastric cancer ranks as the third most common cause of mortality among malignant neoplasms, with 26,380 new cases annually, posing a significant danger to public health.^34^

Numerous independent investigations demonstrated miR-23b down-regulation in gastric cancer tissues, cells, and plasma exosomes.^35^, ^36^, ^37^, ^38^, ^39^ Furthermore, its expression level was positively correlated with disease prognosis, including overall survival, disease-free survival, and survival rate.^35^, ^36^, ^37^^,^^39^, ^40^, ^41^ Conversely, Hu et al^42^ demonstrated via a series of tests that miR-23a/b expression was up-regulated in gastric cancer tissues, functioning as an oncogene that targeted programmed cell death 4 (PDCD4) to block apoptosis in gastric cancer cells, thereby facilitating tumor development. Differences can be ascribed to numerous factors. i) Gene families show that miR-23a/b, though part of the same miR-23∼27∼24 cluster, are located in different chromosomal sites.^43^^,^^44^ Hu’s study evaluated miR-23a/b as a whole, while previous studies focused just on miR-23b, which may behave contradictorily. ii) Research samples are small. Representing the whole context may skew experimental results and affect miR-23b function assessment. iii) DNA methylation and histone modification affect gene expression epigenetically.^45^ The miR-23b gene’s methylation status may affect its expression, affecting target gene regulation and resulting in different functions in different studies. iv) The tumor microenvironment affects expression. Some studies showed that chemotherapeutic drugs affected miR-23b expression,^40^ suggesting that tumor microenvironment factors might alter its function. Cytokines and immune cells in the tumor microenvironment interact with cancer cells, which could activate or inhibit miR-23b along distinct signaling pathways in different studies, leading to uneven functional expression.

miR-23b participates in tumor growth, migration, and other processes. Xian et al^46^ elucidated that miR-23b-3p targeted down-regulation of activated cannabinoid 1 receptor (CB1R) to inhibit Wnt/β-catenin signaling pathway. Moreover, Yan et al^35^ elucidated the function of the circNFATC3/miR-23b-3p/retinoic acid induced 14 (RAI14) axis in gastric cancer cells by biological function assays and subcutaneous xenograft modeling. In 2015, Huang et al^38^ demonstrated that miR-23b reduced tumor development and pluripotency gene expression in gastric cancer cells by targeting the Notch2 receptor or Ets1, influencing tumor bulb hyperstructure via *in vitro* tests. Subsequent *in vivo* tests demonstrated that miR-23b suppressed xenograft tumor proliferation and lung metastasis of SC-M1 gastric cancer cells via the Notch2 pathway.^38^

### Hepatocellular carcinoma

The prevalence of primary liver cancer is rising and is a significant contributor to cancer-related death globally.^47^ Hepatocellular carcinoma (HCC) is the most prevalent type of liver cancer, accounting for roughly 90 percent of cases.^48^

Concerning miR-23b and its isoforms (miR-23b-3p, miR-23b-5p), the majority of research showed its down-regulation in both primary HCC tumors and liquid biopsies (plasma or serum) from cancer patients.^49^, ^50^, ^51^ Conversely, another investigation indicated that miR-23b-3p was increased in HCC tissues compared with the primary tumor and facilitated the proliferation and spread of HCC cells *in vitro* and *in vivo*, contradicting previously published findings.^52^ Conflicting outcomes may be due to these factors: i) Study samples and cell lines are inconsistent. Numerous studies use HCC tissue samples from varied sources and patient demographics, resulting in heterogeneous cell lines that confuse miR-23b′s expression pattern and activity and impede its role assessment. ii) Detection and data analysis methods differ. The differences in technical sensitivity, accuracy, and analytical tools used to quantify miR-23b expression between research influence the understanding of its function. iii) Target genes and regulatory mechanisms vary. The target genes and signaling pathways that miR-23b activates or suppresses vary between studies, resulting in intricate interconnections and functional features. Furthermore, the down-regulation of miR-23b in HCC is significantly correlated with reduced patient survival and adverse clinicopathological characteristics.^51^^,^^53^^,^^54^

While miR-23b-3p and miR-23b-5p are processed from the same pre-miRNA (pre-miR-23b), growing evidence shows that they can exhibit distinct or even opposing functions in cancer. In HCC, miR-23b-5p acted as an oncogene by targeting forkhead box M1 (FOXM1) to arrest the cell cycle and inhibit proliferation.^55^ But the role of miR-23b-3p appears more complex. As reported by Salvi et al,^52^ miR-23b-3p overexpression led to a decrease in urokinase-type plasminogen activator (uPA) and c-mesenchymal–epithelial transition factor (c-MET), which inhibited the migration of HCC cells. He et al^49^ established that miR-23b-3p expression levels were associated with tumor invasiveness, vascular invasion, and lymphatic metastasis. A recent study corroborates these findings, indicating that the inhibition of miR-23b-3p could activate c-MET-induced epithelial–mesenchymal transition to modulate CD44, consequently augmenting tumor cell invasiveness.^50^ Cao et al^53^ discovered that miR-23b-3p affected epithelial–mesenchymal transition by targeting proline-rich tyrosine kinase 2 (Pyk2), influencing HCC migration and invasion. Furthermore, overexpression of miR-23b-3p caused mitochondrial dysfunction and accelerated the switch from mitochondrial respiration to glycolysis in cancer cells, contributing to carcinogenesis, in line with the “Warburg effect” in cancer cells.^56^^,^^57^

### Other tumors of the digestive system

Esophageal cancer (EC) is one of the most prevalent malignant tumors of the digestive system, principally encompassing two histological types: esophageal squamous cell carcinoma and esophageal adenocarcinoma. Most research results demonstrated that miR-23b, as a ts-miR, was weakly expressed in EC cell lines and tissues, and the expression level was substantially connected with cancer development and survival rate.^58^ Conversely, Zhang et al^59^ demonstrated that miR-23b-3p expression was markedly elevated in the tissues of six esophageal squamous cell carcinoma cell lines relative to normal esophageal epithelial cells and directly targeted the tumor suppressor gene early B cell factor 3 (EBF3) to facilitate epithelial–mesenchymal transition, thereby augmenting the invasion and metastasis of tumor cells. The relationship between these divergent conclusions and the isoforms of miR-23b, or the variability in expression across different cell lines, requires validation through additional research.

Cholangiocarcinoma is one of the most frequent primary hepatobiliary malignancies with a poor clinical prognosis, and its 5-year survival rate is less than 15 %.^60^ Sun et al^61^ were the first to recognize LINC00184 as an oncogene associated with cholangiocarcinoma. Using bioinformatics approaches, they observed that cholangiocarcinoma tissues (*n* = 30) showed enhanced expression of LINC00184 and lower expression levels of miR-23b-3p and annexin A2 (ANXA2) relative to standard samples (*n* = 30).^61^ Furthermore, diminished expression of miR-23b-3p was correlated with reduced survival, indicating that miR-23b-3p exerts an inhibitory effect on cholangiocarcinoma progression. Subsequent findings demonstrated that LINC00184 favorably modulated ANXA2 by sponge treatment with miR-23b-3p, thus influencing the proliferation, invasion, migration, and adenine metabolism of cholangiocarcinoma cells.^61^

## Tumors of the reproductive system

### Ovarian cancer

**Figure 2:**
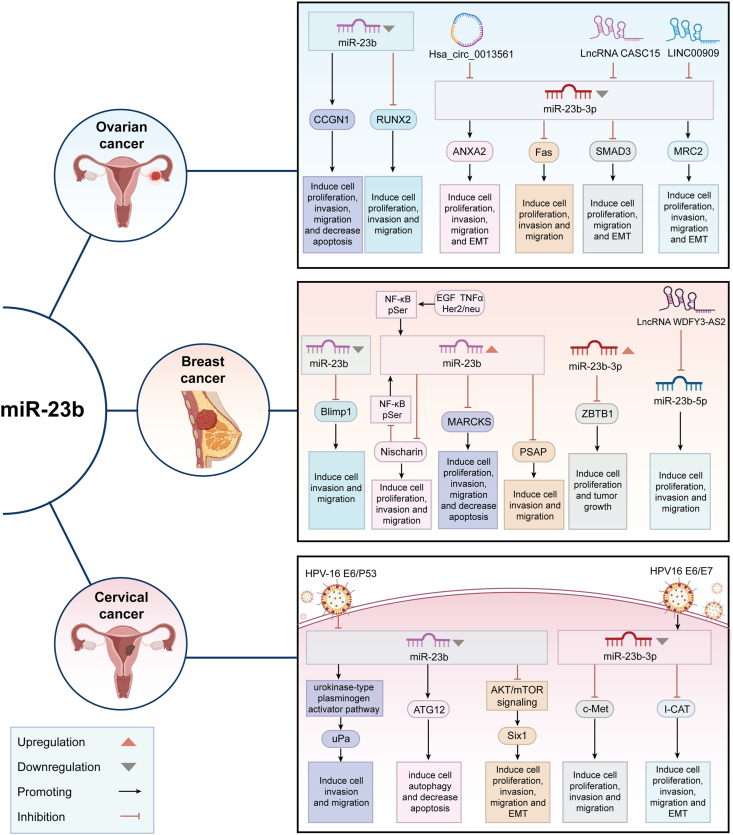
Role and mechanism of miR-23b in reproductive system tumors. Created with BioRender.

Ovarian cancer ranks as the eighth most prevalent disease among women worldwide, representing approximately 3.7% of cases and 4.7% of cancer-related fatalities in 2020.^62^ Initial tests demonstrated that miR-23b was down-regulated in ovarian cancer and junctional tumor tissues relative to standard controls, with a considerable variation in expression based on age and clinical subtype (mucinous versus other kinds).^63^ miR-23b diminished cell proliferation by down-regulating cyclin G1 (CCNG1) and uPA/P70S6K expression, inhibited cell migration and invasion by down-regulating uPA/matrix metallopeptidase 9 (MMP9) expression, and induced apoptosis by down-regulating survivin and B-cell lymphoma-extra-large (Bcl-xL)/uPA expression.^63^ A separate clinical investigation indicated that miR-23b levels in epithelial ovarian cancer were favorably associated with overall survival and disease-free survival, whereas patients with advanced stages and reduced miR-23b expression had a worse prognosis.^64^ Through a comprehensive investigation of the target genes of miR-23b-3p, the researchers established that miR-23b-3p might directly associate with the 3′UTR of Fas gene mRNA, therefore down-regulating Fas protein expression implicated in the regulation of ovarian cancer^65^ (Fig. 2).

### Breast cancer

Breast cancer is the most prevalent malignancy among women globally.^66^ Initial research indicated that miR-23b was significantly expressed in breast cancer tissues and cells, facilitating the growth of breast cancer cells.^67^ Additionally, miR-23b was implicated in breast cancer metastasis. Jin et al^67^ proposed that the expression levels of miR-23b/27b were positively associated with breast cancer invasion and metastasis and unfavorable prognosis. This was accomplished through the down-regulation of the lung metastasis suppressor Nischarin and the up-regulation of the AKT/NF-κB signaling pathway, including HER2/neu (ERBB2), epidermal growth factor (EGF), and tumor necrosis factor-α (TNF-α), all of which were linked to unfavorable breast cancer prognosis^67^^,^^68^ (Fig. 2). Ono et al^69^ found that exosomal transfer of miR-23b from bone marrow inhibited the target gene myristoylated alanine-rich C-kinase substrate (MARCKS), causing breast cancer cells in the metastatic microenvironment to dormancy and reduced CD44 surface expression. However, the tumor microenvironment is complex, and many factors affect miR-23b. Immune cells and their cytokines might affect miR-23b expression,^70^ as might hypoxia^71^ and stromal cell releases.^67^ Thus, while miR-23b promotes breast cancer cell dormancy, its mechanism in complex microenvironments needs further study.

In contrast to findings from certain studies indicating that miR-23b had a cancer-promoting role, Sciortino et al^72^ discovered that in the p130Cas/ErbB2 breast cancer model, miR-23b played an anti-cancer role by down-regulating the expression of the oncogenic factor B lymphocyte-induced maturation protein-1 (Blimp-1). The method of its down-regulation is associated with epigenetic regulation; specifically, the methylation of CpG islands in the regulatory region of the miR-23b gene influences its expression. The divergent functions of miR-23b across several studies may stem from discrepancies in model development. Utilizing diverse cell lines, animal models, and experimental circumstances across numerous studies results in distinct cellular microenvironments for miR-23b. Simultaneously, clinical tissues exhibit heterogeneity. The gene expression and epigenetic alterations of tumor cells vary among patients, and the stage of tumor progression also affects the activity of miR-23b. These factors jointly account for the discrepancies in research outcomes.

It is noteworthy that miR-23b-3p and miR-23b-5p, though derived from the same pre-miR-23b, both exhibit pro-tumorigenic roles in breast cancer via distinct regulatory pathways. miR-23b-5p promotes breast cancer cell migration and invasion by associating with lncRNA WDFY3-AS2,^73^ while miR-23b-3p enhances tamoxifen resistance and aerobic glycolysis by targeting BTB domain containing 1 (ZBTB1).^74^ These findings indicate that the two strands of miR-23b might collaboratively drive breast cancer progression through independent biochemical mechanisms.

### Cervical cancer

Cervical cancer is a prevalent malignant neoplasm among gynecological disorders, with its incidence rate ranking second to breast cancer, posing a significant threat to women’s health.^75^ The expression of miR-23b was modulated by the methylation of its promoter, exhibiting considerable down-regulation in cervical cancer tissues and cell lines.^76^, ^77^, ^78^, ^79^ Furthermore, its expression level was inversely connected with the incidence of precancerous lesions and unfavorable prognosis.^76^, ^77^, ^78^, ^79^ Li and Campos-Viguri et al^77^^,^^78^ demonstrated that decreased miR-23b expression heightened the incidence of cervical cancer in human papillomavirus 16 (HPV16)-positive women and correlated with elevated lesion grade, unfavorable prognosis, and shorter overall survival in patients. Multiple independent studies validated that the overexpression of miR-23b impeded the proliferation, invasion, and migration of cervical cancer cells by down-regulating target genes such as uPa, c-Met, sine oculis homeobox 1 (Six1), and zinc finger E-box-binding homeobox protein 1 (Zeb1), thereby decelerating epithelial–mesenchymal transition^77^, ^78^, ^79^ (Fig. 2).

HPV infection is the primary etiological agent of cervical cancer.^80^ Yeung et al^76^ discovered that the oncogenic HPV-16 E6 protein promoted cervical cancer cell migration by modulating the tumor suppressor p53, down-regulating miR-23b, and enhancing the production of uPA. Hu et al^81^ demonstrated that HPV-16 E6 and E7 enhanced proliferation, migration, invasion, and the epithelial–mesenchymal transition of cervical cancer by modulating the miR-23b-3p/ICAT axis (Fig. 2).

### Head and neck tumors

**Figure 3:**
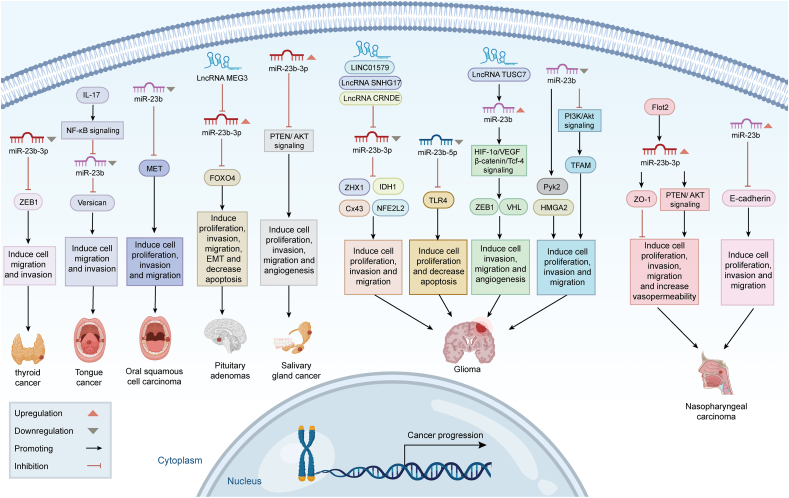
Role and mechanism of miR-23b in neurological and head and neck tumors. Created with BioRender.

Nasopharyngeal carcinoma is a malignant neoplasm of the head and neck that arises from the epithelial cells of the nasopharyngeal mucosa.^82^ Yan et al^83^ discovered that miR-23b-3p expression was elevated in nasopharyngeal carcinoma tissues and that flotillin 2 (FLOT2), a crucial gene for tumor metastasis, facilitated angiogenesis in the tumor microenvironment by targeting exosomal miR-23b-3p to suppress zonula occludens 1 (ZO-1) and activate the phosphatase and tensin homolog (PTEN)/AKT pathway. Moreover, miR-23b-3p stimulated tubule formation in human umbilical vein endothelial cells, increased vascular endothelial cell permeability and migration of tumor cells, and facilitated vascular neovascularization in human umbilical vein endothelial cells.^83^ Notably, miR-23b was implicated in the proliferation, migration, and invasion of nasopharyngeal carcinoma. Wang et al^84^ established that miR-23b down-regulated E-cadherin expression post-transcriptionally via mRNA cleavage, thereby enhancing nasopharyngeal carcinoma cell proliferation and metastasis (Fig. 3).

Salivary adenoid cystic carcinoma constitutes around 10% of salivary gland tumors, making it one of the most prevalent types.^85^ Notwithstanding progress in the gold-standard treatments of surgery and radiation, local recurrence and pulmonary metastases persist in almost one-third of patients.^86^ Hou et al^87^ discovered that exosomal miR-23b-3p was significantly up-regulated in salivary adenoid cystic carcinoma cancer cells relative to normal tissues and that it compromised the integrity of the endothelium barrier by inhibiting the PTEN/AKT pathway and hence facilitated angiogenesis and enhanced tumor dissemination (Fig. 3). Exosomes released by cancer cells have been demonstrated to function as communication mediators, transmitting active chemicals to adjacent stromal cells, influencing recipient cells, and facilitating cancer progression.^88^ Exosomes generated from salivary adenoid cystic carcinoma cells conveyed miR-23b-3p to vascular endothelial cells and were involved in tumor growth, apoptosis, and migration.^87^

Anaplastic thyroid carcinoma is an uncommon yet very aggressive form of thyroid cancer, with over 40% of patients exhibiting distant metastases and a median survival duration of about 3–7 months.^89^ Zhang et al^90^ discovered that the expression level of miR-23b-3p in anaplastic thyroid carcinoma tissues was diminished compared with paracancerous tissues. In contrast, the expression level of ZEB1 was elevated relative to paracancerous tissues, indicating a negative correlation between the two expression levels in anaplastic thyroid carcinoma tissues. Experimental confirmation indicated that the overexpression of miR-23b-3p targeted and down-regulated ZEB1 expression, and inhibited angiogenic mimicry, and diminished the invasive and migratory capabilities of anaplastic thyroid carcinoma cells while concurrently reducing the levels of vascular endothelial growth factor (VEGF), matrix metallopeptidase 2 (MMP2), and MMP9 proteins in the cells^90^ (Fig. 3). The study may offer insights and empirical data for the focused therapy of anaplastic thyroid carcinoma.

Oral squamous cell carcinoma arises from multilayered squamous epithelium and has elevated morbidity and death rates.^47^ Fukumoto et al^91^ discovered that miR-23b expression levels were markedly reduced in oral squamous cell carcinoma specimens, whereas MET was prominently expressed in cancerous tissues. Computer analysis and luciferase reporter gene assays indicated that miR-23b and miR-27b directly targeted the MET oncogene, reducing its expression and inhibiting the migration and invasion of oral squamous cell carcinoma cells^91^ (Fig. 3). Consequently, identifying novel target genes and pathways controlled by the tumor suppressor miR-23b/27b cluster may yield valuable insights for oral squamous cell carcinoma and facilitate the development of innovative treatment options.

Tongue squamous cell carcinoma is a malignant neoplasm arising from the squamous cells of the tongue mucosa and represents the predominant variant of oral squamous cell carcinoma.^92^ Wei et al^93^ found that interleukin-17 (IL-17A) levels were significantly elevated in serum and tumor samples from tongue squamous cell carcinoma patients and positively correlated with tumor metastasis and clinical stage, whereas miR-23b was down-regulated in 93.7% of tongue squamous cell carcinoma tissues and negatively correlated with IL-17A expression (Fig. 3). *In vitro* tests showed that the overexpression of miR-23b suppressed the migratory and invasive capabilities of SCC15 cells.^93^

### Neurologic tumors

Glioma has an annual incidence of 3–6.4 per 100,000, representing roughly 23.3% of all central nervous system tumors and 78.3% of malignant tumors.^47^ Recent findings indicated that miR-23b was present at low levels in glioma tissues and cell lines, and its elevated expression enhanced tumor cell death while inhibiting cell proliferation, migration, and invasion *in vitro*.^94^, ^95^, ^96^ Conversely, several studies suggest that miR-23b functions as a pro-carcinogenic miRNA linked to lncRNA tumor suppressor candidate 7 (TUSC7), ZEB1, and von Hippel-Lindau (VHL) and that its expression level is positively correlated with tumor aggressiveness and WHO grade.^97^, ^98^, ^99^ Consequently, we anticipate robust research with higher sample sizes to elucidate the role of miR-23b in glioma. Moreover, recent findings indicate that VHL modulates the influence of miR-23b on glioma survival and invasion by suppressing hypoxia-inducible factor-1α (HIF-1α)/VEGF and β-catenin/transcription factor 4 (Tcf-4) signaling pathways.^97^ The PI3K/Akt signaling pathway and its downstream effectors, MMP2 and MMP9, participate in the regulatory network of miR-23b and mitochondrial transcription factor A (TFAM) in glioma U251 cells.^96^ It is noteworthy that target molecules, including isocitrate dehydrogenase 1 (IDH1), toll-like receptor 4 (TLR4), high mobility group AT-Hook 2 (HMGA2), and mitochondrial transcription factor A (TFAM), are identified from the specific expression of miR-23b in glioma (Fig. 3), which is anticipated to serve as a pathological diagnostic tool or a molecularly targeted therapy.^94^^,^^95^^,^^100^

Glioblastoma is an intractable primary brain neoplasm arising in the cerebral hemispheres, characterized by a 5-year survival rate of just 5%.^101^ Zhang et al^102^ discovered that LINC01579 functioned as a competitive endogenous RNA (ceRNA) to modulate the expression of nuclear factor erythroid 2-related factor 2 (NFE2L2) by sequestering miR-23b-3p, thereby facilitating the malignant growth of glioblastoma cells. Furthermore, Ge et al^103^ demonstrated that short nucleolar RNA host gene 17 (SNHG17) up-regulated zinc fingers and homeoboxes 1 (ZHX1) by targeting miR-23b-3p, hence enhancing the proliferation and invasion of glioblastoma cells (Fig. 3). These investigations identified miR-23b-3p as a ts-miR implicated in the proliferation, invasion, and migration of glioblastoma, offering potential functional networks and therapeutic targets for the condition.

Pituitary tumors are among the most prevalent intracranial tumors, with an annual incidence of 3.9–7.4 per 100,000, ranking second only to gliomas and meningiomas, and constituting around 15% of all intracranial tumors.^104^ Wang et al^105^ identified miR-23b-3p as a pro-carcinogenic factor controlled by lncRNA MEG3, which is implicated in the proliferation, invasion, migration, and epithelial–mesenchymal transition of pituitary tumor cells. This work demonstrated that miR-23b-3p targeted and down-regulated the expression of forkhead box O4 (FOXO4)^105^ (Fig. 3). Consequently, miR-23b-3p inhibitors may facilitate the restoration of pituitary tumor cell proliferation, invasion, migration, and other functions.

## Tumors of the urinary system

### Prostate cancer

Prostate cancer (PCa) is presently the second most common malignant tumor among men globally and the fifth major cause of cancer-related mortality in males.^106^ miR-23b has been extensively researched in PCa, with all studies corroborating its tumor-suppressive function in this context.^107^, ^108^, ^109^, ^110^, ^111^, ^112^ miR-23b functions as a methylation-silenced ts-miR in PCa, and its tumor-suppressive actions are partially mediated via the regulation of the Src–Akt axis in PCa.^110^ Consequently, the re-expression of miR-23b may facilitate the epigenetic therapy of PCa.

**Figure 4:**
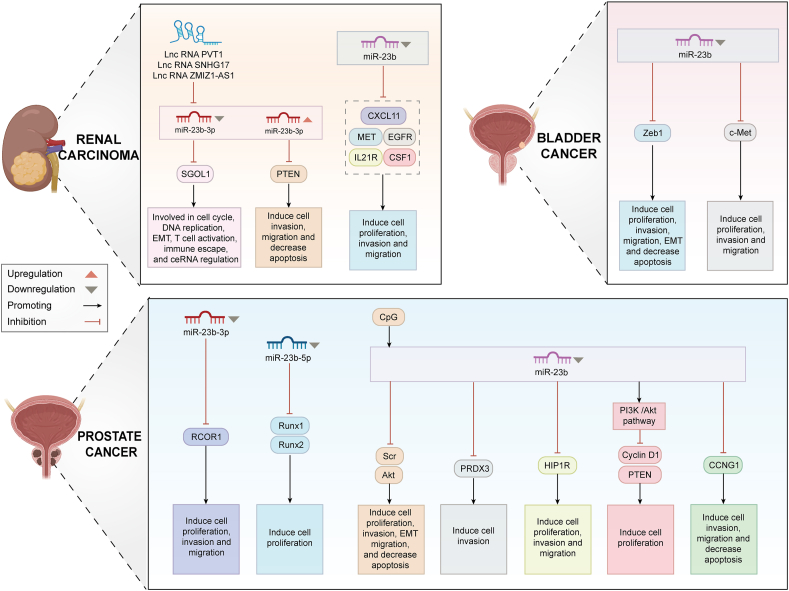
Role and mechanism of miR-23b in urinary system tumors. Created with BioRender.

Numerous experimental studies demonstrated that miR-23b targeted factors, including interleukin-6 receptor (IL-6R), REST corepressor 1 (RCOR1), peroxiredoxin 3 (PRDX3), and PTEN, and was linked to the Janus kinase (JAK)/STAT and PI3K/Akt signaling pathways^108^^,^^109^^,^^113^^,^^114^ (Fig. 4). PTEN belongs to the protein tyrosine phosphatase (PTP) gene family and influences several biological processes.^115^ Tian et al^114^ initially postulated that four miRNAs, including miR-23b, co-regulated PTEN expression in PCa cells and influenced the downstream PI3K/Akt pathway through several regulators, enhancing PCa cell proliferation *in vitro*. Separate research identified a Runx/miRNA interaction axis focused on PTEN-PI3K-AKT signaling, indicating that deleting miRNAs like miR-23b-5p may result in abnormal Runx expression in PCa and influence tumor growth.^107^ He et al^109^ discovered a new miR-23b target, PRDX3, utilizing microarray databases and experimental techniques. PRDX proteins are crucial regulators of cellular antioxidant defense and have been demonstrated to have a role in malignant transformation and therapeutic resistance.^116^ Consequently, miR-23b′s targeting of PRDX3 is believed to have a role in the response of PCa cells to hypoxic stress.

The miR-23b cluster is implicated in migrating and invading PCa tumor cells. In 2012, Ishteiwy et al^117^ identified miR-23b/-27b as a specific inhibitor of critical metastatic processes *in vitro*, encompassing cell invasion, migration, and non-anchored survival. A few years later, the group conducted *in vivo* investigations showing that miR-23b/-27b expression in invasive PCa cells implanted *in situ* markedly restricted their capacity for local invasion and distant metastasis *in vivo*^111^ (Fig. 4).

### Other urological tumors

Renal carcinoma is a malignancy of the genitourinary system that imposes a significant burden on sufferers.^118^ Approximately 85% of renal tumors are identified as renal cell carcinoma, with over 70% exhibiting clear cell histology.^119^ The miR-23b gene is regarded as a potential tumor suppressor in renal carcinoma due to its capacity to block malignant biological processes in renal carcinoma cells. For instance, miR-23b targeted many oncogenes, including EGFR, MET, interleukin 21 receptor (IL21R), C-X-C motif chemokine ligand 11 (CXCL11), and colony-stimulating factor 1 (CSF1), hence inhibiting renal carcinoma proliferation, migration, and invasion.^120^ Yang et al^121^ posited that patients with clear cell renal carcinoma exhibiting diminished miR-23b-3p expression were linked to unfavorable prognoses (overall survival, disease-specific survival, and progression-free interval). Additionally, the SNHG17/PVT1/ZMIZ1-AS1-miR-23b-3p-SGOL1 axis within the ceRNA network might provide novel insights for immunotherapy in patients with clear cell renal carcinoma^121^ (Fig. 4). Nonetheless, one discovery remains contentious in relation to all the aforementioned research. Zaman et al^122^ analyzed 29 samples of renal cell carcinoma and corresponding normal tissues, finding that miR-23b-3p was up-regulated in 15 samples (52%). They demonstrated that this miRNA directly inhibited the apoptotic gene PTEN, and that silencing its expression resulted in an increase in the overall apoptosis rate and a reduction in the invasiveness of the renal carcinoma cell line.^122^ This discrepancy may arise from variations in sample types, quantities, and sources, differences in assay methodologies and techniques, and variations in cellular and tumor microenvironments. Furthermore, miR-23b-3p exhibits inconsistent expression across different samples in this study, and is potentially attributable to individual variability and tumor heterogeneity.

In 2020, an estimated 573,278 individuals globally received a new diagnosis of bladder cancer, with the WHO projecting that prevalence will double by 2040.^47^ Majid et al^123^ noted a substantial down-regulation of miR-23b levels in bladder cancer tissues relative to normal tissues, as determined by patient sample analysis, and elevated miR-23b expression was positively associated with improved overall survival in bladder cancer patients. The reinstatement of this miRNA expression in bladder cancer cell lines suppressed cell proliferation, colony formation, and migration/invasion, and caused G0/G1 cell cycle arrest and death by regulating Zeb1, which facilitated epithelial–mesenchymal transition.^123^ Separate research revealed that suppressing miR-23b/27b cluster expression directly modulated the EGFR and c-Met signaling pathways, promoting cancer cell proliferation, migration, and invasion in bladder cancer^124^ (Fig. 4). This suggested that miR-23b could serve as a viable treatment approach for bladder cancer, offering insights for more pharmacological investigation.

### Pulmonary neoplasms

Pulmonary neoplasms are considered one of the leading causes of cancer-related deaths worldwide. Non-small cell lung cancer is the most common subtype, accounting for about 80%–85% of all lung cancers.^125^ A preliminary investigation by Han et al^126^ examined miR-23b expression in 57 pairs of pulmonary neoplasm and adjacent non-cancerous tissues, revealing that miR-23b expression was markedly reduced in malignant tissues, and this down-regulation was correlated with diminished overall survival and disease-free survival in patients. Liu et al^127^ analyzed miR-23b levels in 127 non-small cell lung cancer and normal tissues using quantitative PCR, reaching the same finding. These findings were similarly corroborated in quantitative PCR experiments comparing pulmonary neoplasm cell lines with normal cells.^126^^,^^128^ Consequently, miR-23b functions as a ts-miR in pulmonary neoplasm.

miR-23b was demonstrated to target myeloid leukemia 1 (MCL-1), CCNG1, and other pathways,^126^^,^^129^ influencing biological behaviors such as proliferation, migration, invasion, and epithelial–mesenchymal transition in non-small cell lung cancer cells. Moreover, the JAK/STAT and Wnt/β-catenin pathways were shown to contribute to many tumor cells' proliferation and malignant transformation, including those in pulmonary neoplasms.^129^ In summary, miR-23b targets several mRNAs to elicit anti-cancer effects in pulmonary neoplasms, especially in non-small cell lung cancer. miR-23b may serve as a promising biomarker for pulmonary neoplasm and is anticipated as a potential instrument for targeted therapy in non-small cell lung cancer.

## miR-23b in tumor treatment resistance

### 5-Fluorouracil and oxaliplatin

5-Fluorouracil plus oxaliplatin resistance is a significant barrier in the treatment of CRC.^130^ Xian et al^131^ analyzed miR-23b-3p levels in 5-fluorouracil-sensitive and -resistant CRC tissues using quantitative reverse transcription PCR and discovered that it was less abundant in the sensitive tissues. Subsequent *ex vivo* investigations demonstrated that the urothelial carcinoma-associated 1 (UCA1)/miR-23b-3p/zinc finger protein 281 (ZNF281) axis played a role in the modulation of apoptosis, autophagy, and tumor sensitivity to 5-fluorouracil in CRC cells.^131^ Chen et al^132^ demonstrated that miR-23b-3p expression was reduced in oxaliplatin serum and drug-resistant CRC cells, whereas the expression of high mobility group box 2 (HMGB2), a direct target of this miRNA, enhanced the chemosensitivity of drug-resistant CRC cells to oxaliplatin. Conversely, Gasiulė et al^133^ discovered that miR-23b was significantly up-regulated in oxaliplatin-resistant CRC cell lines (HCT-Oxa-c and HCT-Oxa-p), and its suppression led to a 3.5-fold enhancement in the sensitivity of HCT-Oxa-c cells to oxaliplatin. The researchers further demonstrated that miR-23b had a role in modulating the intricate epithelial–mesenchymal transition, which enhanced resistance to oxaliplatin in CRC cells.^133^ The authors hypothesized in the discussion section that miR-23b might need to preserve a balance in the cellular epithelial–mesenchymal transition to develop and sustain oxaliplatin resistance.

### Sorafenib

Sorafenib, a multikinase inhibitor, is a first-line therapy for advanced HCC.^134^ Jing et al^54^ identified the expression of miR-23b-3p by creating a sorafenib-resistant Hep3B cell model and observed that it exhibited low expression across all HCC tissues, with the lowest levels seen in sorafenib-resistant HCC tissues. A mouse model was developed to verify that short nucleolar RNA host gene 16 (SNHG16) suppressed miR-23b-3p expression by sequestering early growth response gene-1 (EGR1), which enhanced survival, autophagy, and the suppression of sorafenib-resistant HCC cells.^54^ Recent research indicated that miR-23b-3p enhanced autophagy by targeting autophagy-related 12 (ATG12) and fostered glutamine dependency by targeting glutaminase 1 (GLS1), thus providing a cytoprotective impact in sorafenib-resistant HCC.^135^

### Flutamide

Flutamide, the first non-steroidal anti-androgen, is extensively utilized in managing advanced PCa, but survival rates are diminished when administered as monotherapy.^136^ Pimenta et al addressed this problem and found that when miR-23b transfection was used in conjunction with flutamide, it sensitized PC-3 cells to the effects of flutamide, increased apoptosis, and reduced CCNG1 oncogene expression.^112^ This implies that miR-23b agonists in combination with flutamide could be a novel approach for treating depot-resistant PCa.

### Relationship of miR-23b with lncRNA/circRNA and downstream target genes in tumors

lncRNAs and circRNAs significantly regulate many malignancies by interacting with miR-23b and its isoforms. Data were gathered and categorized by lncRNAs, circRNAs, cancer species, target miRNAs, genes, and other relevant factors (Table 1; Fig. S4). TUSC7, SNHG17, and UCA1 are the most extensively researched lncRNAs concerning miR-23b and its isoforms. The circRNAs pertinent to this domain are Hsa_circ_0039930, Hsa_circ_0021727, and Hsa_circ_0013561. Gastric cancer, glioma, and CRC are this domain’s most extensively researched cancer types.

**Table 1:** lncRNAs and circRNAs that bind to miR-23b and the target genes in tumors.

Cancer	lncRNA/circRNA	miRNA	Target genes	Pathway/process affected	*In vitro*/*in vivo*	Cell type	Animal species	References
Gastric cancer	Hsa_circ_0039930	miR-23b-3p	RAI14	PI3K/AKT signaling pathway, cell proliferation, cell invasion, apoptosis	*In vitro*, *in vivo*	GES-1 and GC cell lines	Male BALB/c nude mice	Yan et al (2021)^35^
Gastric cancer	lncRNA MALAT1	miR-23b-3p	ATG12	Autophagy, chemoresistance	*In vitro*, *in vivo*	SGC7901 and BGC823	Female athymic nude mice	Hu et al (2017)^41^
Gastric cancer	lncRNA SNHG17	miR-23b-3p	Notch2	Drug resistance, cell differentiation, apoptosis	*In vitro*, *in vivo*	HGC-27, AGS and GES-1	5-week-old BALB/C-A nude mice	Huang et al (2022)^144^
Gastric cancer	lncRNA DLEU2	miR-23b-3p	Notch2	Notch signaling pathway, cell proliferation, cell migration, cell invasion, apoptosis	*In vitro*	GES-1 and GC cell lines	/	Li et al (2021)^145^
Gastric cancer	lncRNA UCA1	miR-23b-3p	IL6R, BCL2 and HSP90B1	Cell proliferation, cell migration, and cell invasion	*In vitro*	SGC-7901	/	Zhang et al (2021)^146^
Gastric cancer	lncRNA TUSC7	miR-23b-3p	/	Tumor cell growth inhibition, cell proliferation regulation	*In vitro*, *in vivo*	AGS and MKN-45 cells	4–6 weeks old male BALB/c nude mice	Qi et al (2015)^36^
CRC	lncRNA UCA1	miR-23b-3p	ZNF281	Autophagy, chemoresistance, apoptosis	*In vitro*, *in vivo*	SW480, SW620, and 293T	Nude mice	Xian et al (2020)^131^
CRC	lncRNA NEAT1	miR-23b-3p	KLF3	Cell proliferation, cell migration, cell invasion, apoptosis, and cell cycle regulation	*In vitro*	HT29, HEK293	/	Hu et al (2022)^147^
CRC	lncRNA TUSC7	miR-23b	PDE7A	Cell proliferation, apoptosis, cell migration, cell invasion, EMT	*In vitro*	LoVo, SW116, CaCo2, HCT-116, SW480 and NCM460	/	Hao et al (2020)^148^
CRC	lncRNA TUSC7	miR-23b	/	Oxidative stress regulation, M2 polarization of macrophages, cell proliferation, cell migration, and cell invasion	*In vitro*, *in vivo*	CT26 cell line	Male BALB/c mice	Ye et al (2023)^149^
Esophageal cancer	Hsa_circ_0021727	miR-23b-5p	/	Cell proliferation, cell migration, cell invasion, and NF-κB signaling pathway activation	*In vitro*, *in vivo*	ESCC cell lines	Male BALB/c nude mice	Meng et al (2023)^19^
Cholangiocarcinoma	LINC00184	miR-23b-3p	ANXA2	Cell proliferation, cell migration, cell invasion, and adenine metabolism	*In vitro*, *in vivo*	KMBC, HuCCT1, QBC939 and HIBEC	Mice	Sun et al (2021)^61^
HCC	lncRNA RP11-422N16.3	miR-23b-3p	DMGDH	Cell proliferation, apoptosis, EMT	*In vitro*	HepG2, SMMC-7721, MHCC97-H, HCCLM3 and L02	/	Sun et al (2019)^150^
HCC	lnc-PLA2G4A-4	miR-23b-3p	Versican	Cell proliferation, cell migration, cell invasion, EMT, AKT/p21 signaling pathway, p-EGFR/p-AKT signaling pathway	*In vitro*	SNU398,SNU449,SK-Hep-1,Hep3B,HepG2/C3A and HEK-293T	/	Xiong et al (2023)^151^
HCC	lncRNA SNHG16	miR-23b-3p	EGR1	Cell proliferation, apoptosis, autophagy, and drug resistance	*In vitro*	The Hep3B cells	/	Jing et al (2020)^54^
HCC	lncRNA FAM66C	miR-23b-3p	KCND2	Cell proliferation, cell migration, cell invasion, EMT, glycolysis	*In vitro*, *in vivo*	HIBEpic cells and ICC cell lines	4–6 weeks old BALB/C male nude mice	Lei et al (2021)^152^
Breast cancer	lncRNA WDFY3-AS2	miR-23b-5p	/	Cell proliferation, cell migration, and cell invasion	*In vitro*	MCF10A MCF7,	/	Aisikaer et al (2022)^73^
						BT474, MDA-MB-468, BT549		
Endometrial carcinoma	lncRNA TUSC7	miR-23b	/	Cell proliferation, cell cycle, apoptosis, chemotherapy sensitivity	*In vitro*	ESC, HEC1A	/	Shang et al (2017)^153^
Ovarian cancer	Hsa_circ_0013561	miR-23b-3p	ANXA2	Cell proliferation, cell migration, cell invasion, EMT	*In vitro*, *in vivo*	SKOV3, ES-2, OVCAR-3, A2780 and IOSE80	Nude mice	Lv et al (2023)^141^
Ovarian cancer	lncRNA CASC15	miR-23b-3p	SMAD3	EMT, cell migration, cell invasion, TGF-β/SMAD3 signaling pathway	*In vitro*, *in vivo*	SKOV3, OVCAR-3, ES-2, A2780 and HEK-293T	NOD/SCID mice	Lin et al (2022)^154^
Ovarian cancer	LINC00909	miR-23b-3p	MRC2	Cell proliferation, cell migration, cell invasion, EMT	*In vitro*	SKOV3, CAOV4, EFO21	/	Yang et al (2021)^155^
Pituitary adenoma	lncRNA MEG3	miR-23b-3p	FOXO4	Cell proliferation, apoptosis, EMT	*In vitro*	GH3 and MMQ	/	Wang et al (2021)^105^
Glioma	lncRNA SNHG17	miR-23b-3p	ZHX1	Cell proliferation, cell migration, and cell invasion	*In vitro*	A172 and DK-MG	/	Ge et al (2023)^156^
Glioma	lncRNA CRNDE	miR-23b-3p	IDH1	Cell proliferation, cell migration	*In vitro*	U-118MG and U251	/	Chen et al (2023)^94^
Glioma	lncRNA SNHG17	miR-23b-3p	ZHX1	Cell proliferation, cell migration, and cell invasion	*In vitro*	NHA and human glioma cell lines H4, A172, U251 and LN229	/	Ge et al (2020)^103^
Glioma	LINC01579	miR-23b-3p	NFE2L2	Cell proliferation, cell migration, and cell invasion	*In vitro*	U87, U251 and HA	/	Zhang et al (2023)^102^
Glioma	lncRNA TUSC7	miR-23b	/	Cell proliferation, apoptosis, cell migration, and cell invasion	*In vitro*	U251, U87, and HEK 293T cells	/	Shang et al (2016)^99^
Renal cancer	SNHG17/PVT1/ZMIZ1-AS1	miR-23b-3p	SGOL1	Cell cycle, DNA replication, EMT, T cell activation, immune escape, ceRNA regulation	*In vitro*	HK-2 and clear cell renal carcinoma cell lines SW839, RCC-4, 769-P, A498, Caki1, 786-O and OS-RC-2	/	Yang et al (2024)^121^
NSCLC	lncRNA SNHG17	miR-23b-3p	/	Cell proliferation, cell migration, and cell cycle regulation	*In vitro*	16HBE and NSCLC cell lines	/	Ha et al (2019)^157^
NSCLC	lncRNA CCAT2	miR-23b-5p	FOXC1	Cell proliferation, cell migration	*In vitro*	BEAS-2B and lung adenocarcinoma cells	/	Hu et al (2019)^128^
NSCLC	lncRNA KTN1-AS1	miR-23b	DEPDC1	Cell proliferation, apoptosis, cell migration, cell invasion, EMT	*In vitro*, *in vivo*	BEAS-2B, NSCLC cells	5-week-old male nude mice	Liu et al (2020)^127^

Abbreviations: SMAD3, SMAD family member 3; CRNDE, colorectal neoplasia differentially expressed; IDH1, isocitrate dehydrogenase (NADP (+)) 1; ZHX1, zinc fingers and homeoboxes 1; PVT1, human plasmacytomvariant translocation 1; ZMIZ1-AS1, ZMIZ1 Antisense RNA 1; CRC, Colorectal cancer; ESCC, esophageal squamous cell carcinoma; NSCLC, non-small cell lung cancer; EMT, epithelial–mesenchymal transition.

### Current miRNA therapy clinical trials and RNA technology potential

We systematically reviewed completed and ongoing clinical studies of miRNA therapies (Table 2) by searching the ClinicalTrials.gov database. These research data provide critical evidentiary support for the clinical application of miRNA therapies.

**Table 2:** Studies of miRNA therapy from ClinicalTrials.gov.

Study title	Study status	Drug	MicroRNA	Registration No.
A multicenter phase I study of MRX34, MicroRNA miR-RX34 liposomal injection	TERMINATED	MRX34	miR-34a	NCT01829971
Pharmacodynamics study of MRX34, MicroRNA liposomal injection in Melanoma patients with biopsy accessible lesions	WITHDRAWN	MRX34	miR-34a	NCT02862145
Efficacy, safety, and tolerability of Remlarsen (MRG-201) following intradermal injection in subjects with a history of Keloids	COMPLETED	Remlarsen	miR-29b	NCT03601052
Study of ATX-01 in participants with DM1	RECRUITING	ATX-01	miR-23b	NCT06300307
Safety, tolerability, pharmacokinetics, and pharmacodynamics of MRG-110 following intradermal injection in healthy volunteers	COMPLETED	MRG-110	miR-92a	NCT03603431
First-in-human study of INT-1B3 in patients with advanced solid tumors	TERMINATED	INT-1B3	miR-193a-3p	NCT04675996
Safety study of SPC3649 in healthy men	COMPLETED	SPC3649	miR-122	NCT00688012
MesomiR 1: A phase I study of TargomiRs as 2nd or 3rd line treatment for patients with recurrent MPM and NSCLC	COMPLETED	TargomiRs	miR-16	NCT02369198
PRISM: Efficacy and safety of Cobomarsen (MRG-106) in subjects with Mycosis Fungoides who have completed the SOLAR study	TERMINATED	Cobomarsen	miR-155	NCT03837457
SOLAR: Efficacy and safety of Cobomarsen (MRG-106) vs. active comparator in subjects with Mycosis Fungoides	TERMINATED	Cobomarsen	miR-155	NCT03713320
Safety, tolerability and pharmacokinetics of MRG-106 in patients with Mycosis Fungoides (MF), CLL, DLBCL or ATLL	COMPLETED	Cobomarsen	miR-155	NCT02580552

As precision medicine advances, RNA technology is also making progress. siRNAs can use degradable polymer-carriers to pinpoint gene silencing and boost tissue regeneration in regenerative medicine.^137^ Nanomaterials enhance RNA delivery while reducing immunogenicity.^138^ Integrating biomedical engineering, nanotechnology, and other multidisciplinary domains will lead to breakthroughs of tailored RNA therapeutic solutions, boosting the global RNA therapeutics market.^139^^,^^140^ We expect RNA technology will focus on tailored delivery, novel materials, and multimodal medicines to change illness treatment.

## Discussion

The articles included in this review exemplify the forefront and current advancements in global cancer research concerning miR-23b. We produced and evaluated miR-23b expression in malignancies, normal tissues, cells, and liquid biopsy specimens. miR-23b is up-regulated or down-regulated during tumor progression and directly correlated with disease prognosis, as shown by survival rates, recurrence, metastasis, and clinicopathological characteristics. Specifically, miR-23b acts as an onco-miR in malignancies, such as nasopharyngeal carcinoma and breast cancer, while demonstrating ts-miR characteristics in CRC, cholangiocarcinoma, ovarian cancer, and glioblastoma.

miR-23b plays two important roles in cancer and is involved in several tumor cell processes. Target genes, signaling pathway networks, and the tumor microenvironment control its function. In hormone-responsive breast cancer, estrogen via estrogen receptor β (ERβ) regulated miR-23b to promote its accumulation, thereby facilitating tumor growth through effects on the tumor microenvironment.^141^ In gastric cancer, microenvironmental changes like *H. pylori* infection down-regulated miR-23b-3p, resulting in inadequate CB1R inhibition and over-activation of the Wnt/β-catenin pathway, promoting tumor progression.^46^ As “molecular sponges”, ceRNAs, lncRNAs, and circRNAs compete with miR-23b in tumor microenvironments. MEG3 overexpression in pituitary adenoma lowered miR-23b-3p and FOXO4 interaction, increasing apoptosis and delaying progression.^105^ High hsa_circ_0013561 ceRNA levels competed with miR-23b-3p to lower ANXA2 and boost ovarian cancer cell proliferation and migration.^142^

miR-23b, a promising biomarker linked to treatment resistance, is expected to be a key cancer treatment target. Studies showed that miR-23b affected cytokine and autophagy pathways to combat multidrug resistance in chemotherapy-resistant cells, such as CRC, PCa, and HCC.^54^^,^^112^^,^^133^ Thus, clinicians can analyze patients' miR-23b levels before treatment, predict treatment responses, and tailor cancer therapy to improve efficacy and reduce side effects.

However, miR-23b faces many challenges in translating basic research into clinical application, with poor tumor selectivity and delivery efficiency being the main issues.^143^ As it regulates multiple target genes, miR-23b may induce immunosuppression or drug resistance while offering therapeutic benefits. Although lipid nanoparticle-based delivery technologies have improved miR-23b bioavailability, off-target effects still pose risks to non-tumor tissues. To address this, future research should enhance targeting specificity by integrating multi-database prediction (*e.g.*, TargetScan, miRDB) with cross-species conservation analysis to screen high-confidence targets, validating findings across diverse cell models (primary cells, patient-derived xenografts, organoids), and combining pathway enrichment with functional rescue assays (*e.g.*, CRISPR-mediated introduction of miR-23b-resistant target gene mutants) to clarify the contribution of key targets to specific signaling pathways like Wnt/β-catenin and PI3K/AKT. These strategies will deepen mechanistic understanding and facilitate the development of precise miRNA mimics or inhibitors with balanced therapeutic efficacy and minimized off-target impacts.

In conclusion, miR-23b is heterogeneously expressed and functionally dual in cancers, closely linked to prognosis, and has biomarker and therapeutic potential. Mechanistic investigations and delivery systems must be improved to assist clinical translation.

## CRediT authorship contribution statement

**Xinyu Cai:** Writing – review & editing, Writing – original draft, Data curation, Conceptualization. **Xueer Zheng:** Writing – review & editing, Writing – original draft. **Zhenru Wang:** Writing – original draft, Visualization. **Jiahua Mao:** Writing – original draft, Data curation. **Minhe Shen:** Supervision, Resources, Funding acquisition. **Shanming Ruan:** Writing – review & editing, Supervision.

## Funding

This work was supported by the 10.13039/501100001809National Natural Science Foundation of China (No. 82274597).

## Conflict of interests

The authors declared no competing interests.
