# Supplementary material for: MicroRNA-23b: Roles, functions and mechanisms in tumor
Source: Genes Dis. 2025 Sep 18;13(4):101853. doi: 10.1016/j.gendis.2025.101853 (PMC13058972; doi:10.1016/j.gendis.2025.101853)
Supplement: Multimedia component 1 [file mmc1.docx]

**SUPPLEMENTARY MATERIAL**

**MicroRNA-23b: Roles, functions and mechanisms in tumor**

**Supplementary Figure**

**Supplementary Figure 1** - The mRNA sequence of miR-23b from NCBI. (National Center for Biotechnology Information (nih.gov)).

**Supplementary Figure 2** - Flowchart of the screening process.

**Supplementary Figure 3** - Analysis of research hotspots and trends of miR-23b. (A) Network relationship diagram for keyword co-occurrence; (B) Keyword citation bursts.

**Supplementary Figure 4** - Sankey diagram.

**Supplementary Table**

**Supplementary Table 1 -** Information of miR-23b from NCBI. (National Center for Biotechnology Information (nih.gov)).

**Supplementary Figure 1** - The mRNA sequence of miR-23b from NCBI. (National Center for Biotechnology Information (nih.gov)).

**
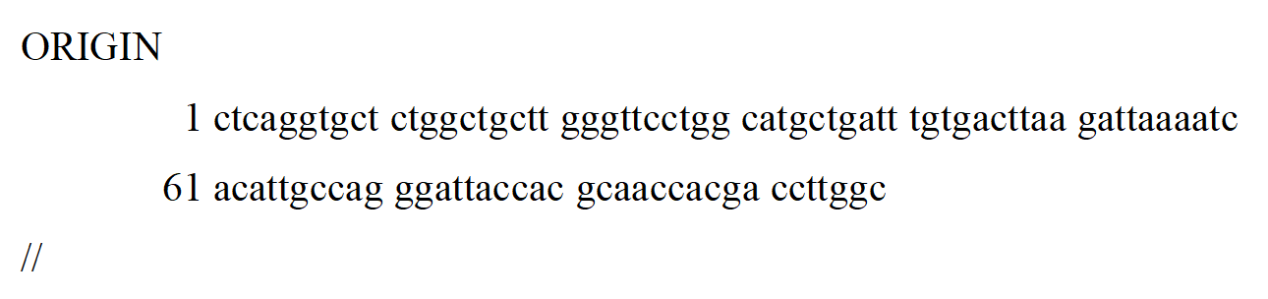
**

**Supplementary Figure 2** - Flowchart of the screening process.


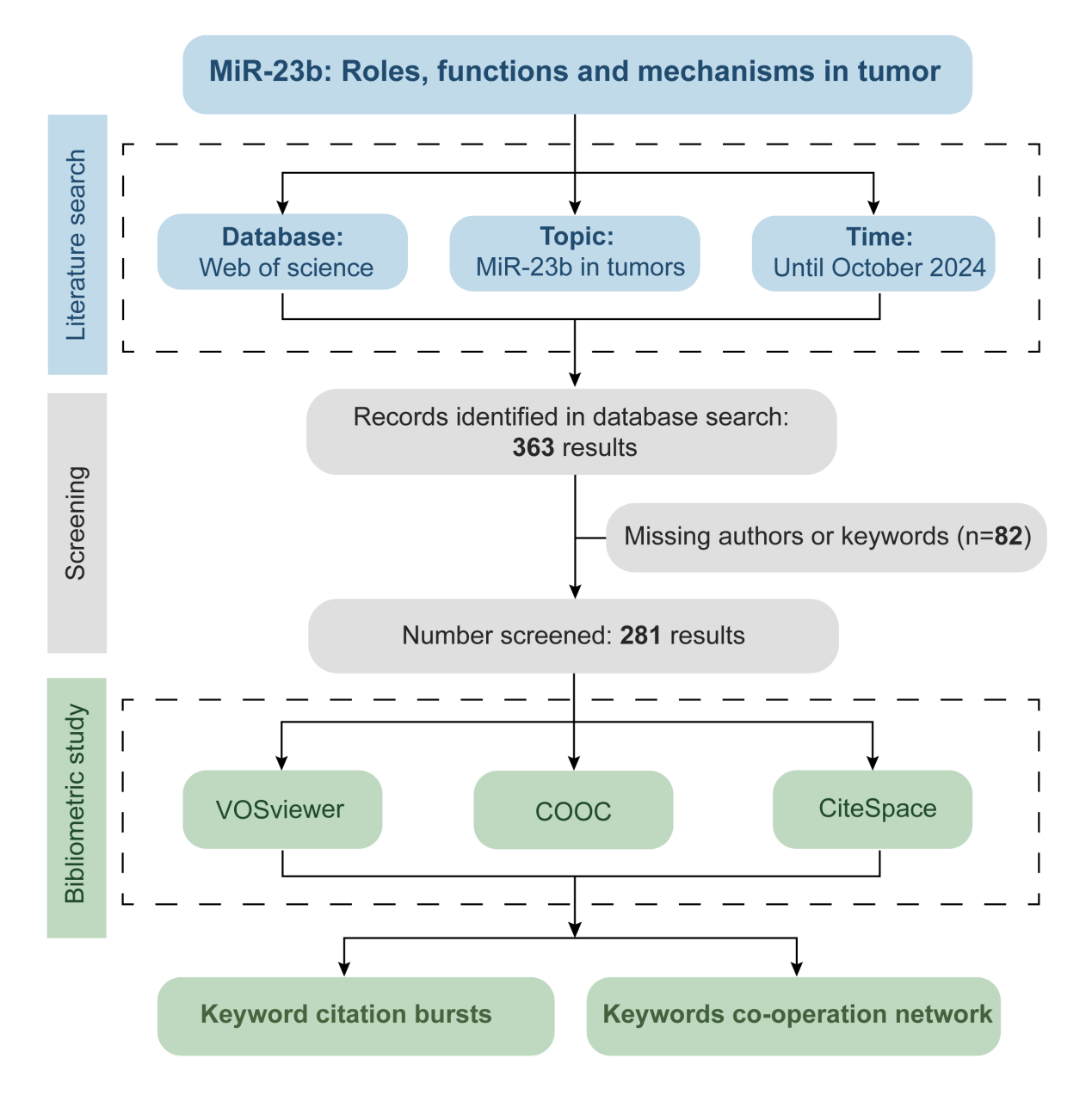


**Supplementary Figure 3 -** Analysis of research hotspots and trends of miR-23b. (A) Network relationship diagram for keyword co-occurrence; (B) Keyword citation bursts.


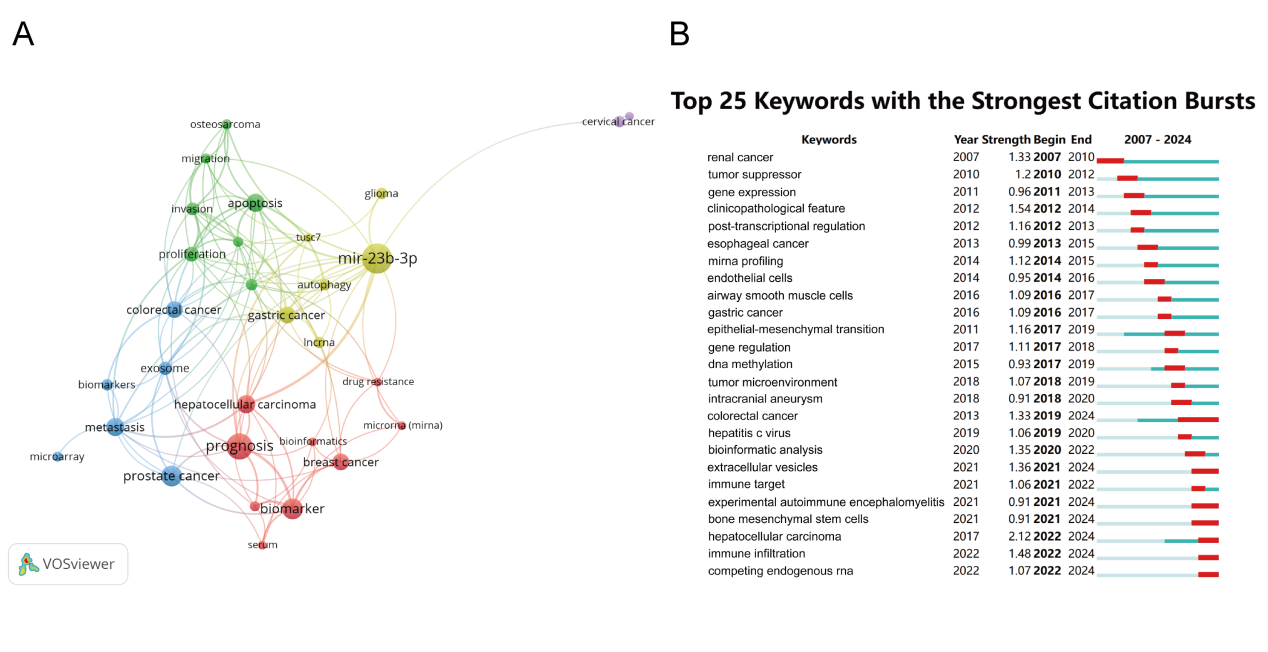


**Supplementary Figure 4** - Sankey diagram.

**
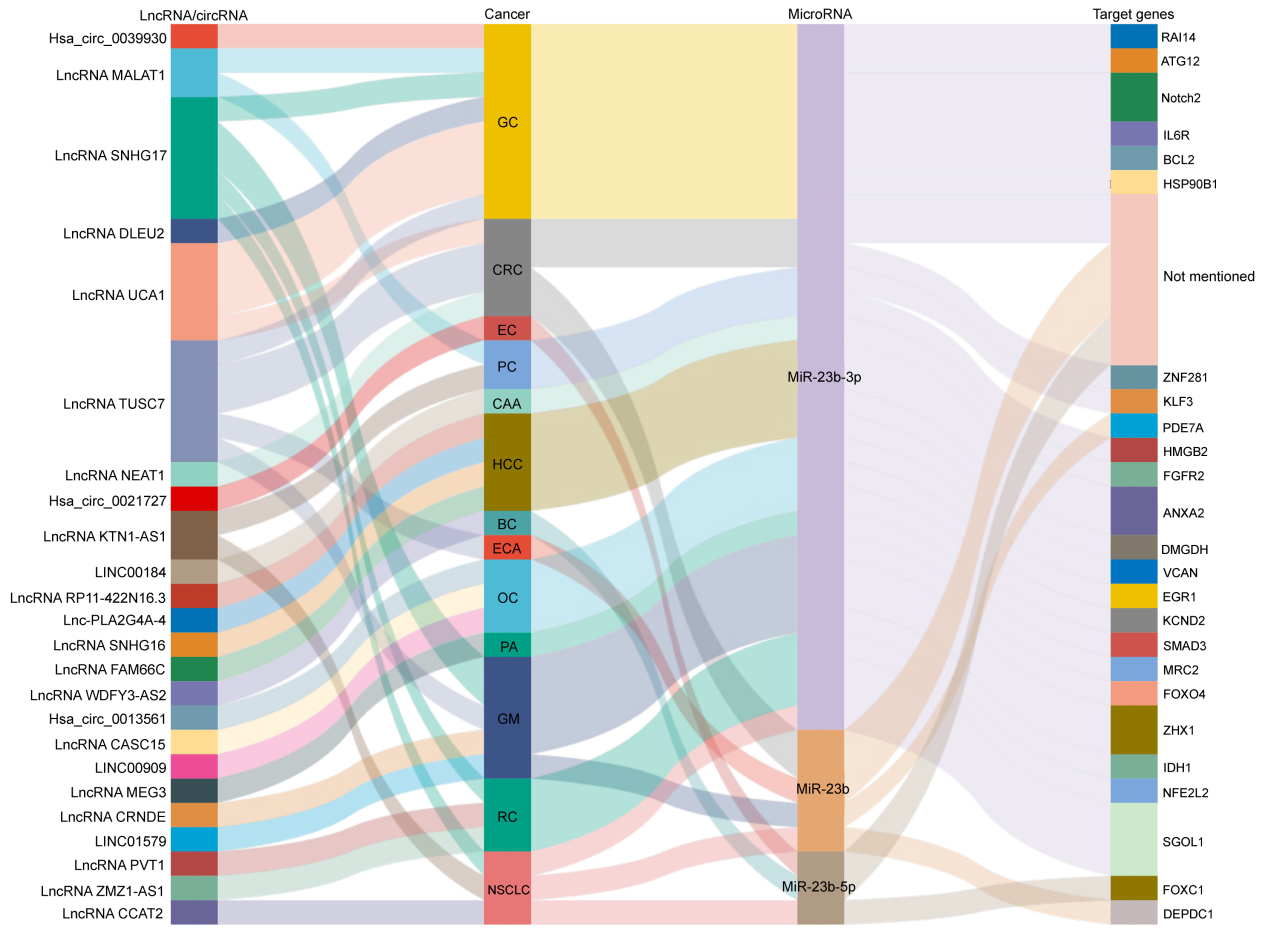
**

**Supplementary Table 1** - Information of miR-23b from NCBI. (National Center for Biotechnology Information (nih.gov)).

| GENE | MIR23b |
| --- | --- |
| Gene ID | 407,011 |
| Organism | Homo sapiens |
| Also known as | MIRN23B; hsa-mir-23b; miRNA23B; mir-23b |
| Location | Chromosome 9, NC_000009.12 (95,085,208-95,085,304) |
| Length | 97 nt |
| Exon count | 1 |
